# Driven or lacking access: Integration types as a subdimension of the affect consciousness construct

**DOI:** 10.3389/fpsyg.2023.968737

**Published:** 2023-02-15

**Authors:** Astrid Gravdal Vølstad, Maria Ingunnsdatter Salas, Ole André Solbakken

**Affiliations:** ^1^Department of Social Sciences and History, Volda University College, Volda, Norway; ^2^Department of Psychology, Faculty of Social Sciences, University of Oslo, Oslo, Norway

**Keywords:** integration types, affect integration, interpersonal problems, assessment, construct validation, individual differences, affective functioning, emotion regulation

## Abstract

**Introduction:**

This article examines integration types as a sub-dimension of the affect consciousness construct to account for individual differences in how problems with the experience and expression of affects manifest. The two integration types driven and lack of access describe prototypical ways of experiencing and expressing affect, differentiating between problems characterized by too much or too little affective mobilization.

**Methods:**

Archival data from a non-clinical sample (n = 157) was used to examine the validity and reliability of integration type scales from the Affect Integration Inventory (AII 2.0). Internal structure was assessed through confirmatory factor analyses (CFAs) by structural equation modelling. Nomological validity was examined through tests of patterns of hypothesized associations between integration types across various affects and specific types of interpersonal problems (as measured by the Inventory of Interpersonal Problems; IIP-64).

**Results:**

CFAs indicated acceptable fit for the different integration type scales and overall construct structure. Distinct sinusoidal patterns of correlations between integration types and interpersonal problems were found for the various affects examined. All correlation patterns had good fit (GoF ≥ 0.87), with significant differences in magnitude between peak and low point correlations.

**Discussion:**

We conclude that differences in prototypical ways of experiencing and expressing affects can be assessed easily, quickly, and reliably, have theoretically consistent intra-domain relationships and valid structural psychometric properties, are robustly related to interpersonal functioning in general, and are systematically and differentially related to specific and theoretically hypothesized interpersonal problem types.

## Introduction

Affect consciousness (AC) is the operationalization of affect integration (Tomkins, 1962; Krystal, 1974; Monsen et al., 1989; Solbakken et al., 2011a; Stolorow et al., 1995). It is usually defined as the functional integration of affect in cognition, motivation, and behavior (Monsen et al., 1996; Monsen and Monsen, 1999; Solbakken et al., 2011b). The concept refers to how we experience, relate to, manage, and express our affects, and is considered an essential factor for wellbeing, mental health, and psychological functioning. The AC-construct as a way of measuring affect integration was developed in the early eighties as a part of a naturalistic psychotherapy study on patients with personality disorders (Monsen et al., 1989). AC was originally operationalized as degrees of awareness, tolerance, non-verbal and conceptual expression for each of 9 discrete affects. A semi-structured interview (the Affect Consciousness Interview; ACI) and separate rating scales (the Affect Consciousness Scales; ACSs) were developed to assess these aspects of affect integration and systematically tested in several studies (e.g., Monsen et al., 1996; Solbakken et al., 2011a). A specific AC treatment model (ACT) was developed and tested (e.g., Monsen et al., 1995; Monsen and Monsen, 2000) and later a self-report instrument for more time-efficient assessment of AC, i.e., the Affect Integration Inventory (AII; Solbakken and Monsen, 2013) was constructed and tested in several studies (e.g., Solbakken et al., 2017; Frederiksen et al., 2021a,b, 2022). As mentioned, AC can formally be differentiated from affect integration as the operationalized measurement of the latter. However, for all practical purposes the two terms can be used interchangeably. Thus, for the sake of simplicity and ease of reading, we will use the term affect integration in the following when referring to the processes in question.

According to affect integration theory (Solbakken et al., 2011b), affects have different inherent functions; each have their own informational value, and motivate us for specific adaptive behaviors (Izard, 1991, 2007). Affect integration is posited to be central to the ability to understand and make use of the adaptive signal function of affects, which in turn guide the individual toward adequate adjustment to the environment, both in intrapersonal and interpersonal situations.

When examining affect integration, researchers investigate whether individuals have high or low levels of integration affect for a given affect. However, two central variants of low affect integration are commonly not differentiated in assessment, i.e., problems characterized by experiencing an affect too intensely (and as a consequence being driven by its impulses or action tendencies) and problems with experiencing too little of it (thereby lacking access to the adaptive signal- and motivational properties of the affect in question), even though these problems tend to differ substantially at the experiential and behavioral levels. The present study therefore examines these particular subdimensions of affect integration, i.e., integration types. Using specific and separate scales from the Affect Integration Inventory constructed to assess these sub-dimensions (Solbakken and Monsen, 2013), we test the validity of a model of affect integration that systematically distinguishes between being driven by and lacking access to a number of specific affects.

This study aims to examine integration types through:

(1) Investigating their conceptual soundness using confirmatory factor analysis (CFA), and
(2) Examining the patterns of associations between integration types and specific interpersonal problems for testing nomological validity.

### Integration types

Essentially, integration types thus refer to two prototypical modes in which affects can be maladaptively experienced and communicated: being *driven* by the affect and *lacking access* to it. These integration types constitute prototypical, problematic ways of experiencing and expressing specific affects (e.g., being driven by anger). Being *driven* by an affect is characterized by being overwhelmed by the affect activation, including uncontrolled or unregulated acting on the impulses inherent in the affect. This is often displayed through non-verbal and verbal expressions and actions that seem impulsive or uncontrolled, such as an anger outburst.

*Lacking access* to an affect denotes the opposite; the impulses and motivational elements of the affect are unavailable to the individual and are often converted to other (often maladaptive) experiential states in which the intensity and/or information in the affect become distorted. For instance, anger or sadness may turn into resignation and hopelessness. Typically, if a person states that they never experience a specific affect or feeling, this may suggest that the access to the affect is diminished.

Conceptually, the integration types are similar to other constructs such as the window of tolerance framework (Siegel, 2012), emotion regulation strategies (Gross and John, 2002; Gross, 2015) and alexithymia (primarily lack of access; Lesser, 1981; Bagby et al., 1994). What primarily separates integration types from other similar concepts is the emphasis in affect integration theory and research on the importance of differentiating between affects. Affects are thus posited to constitute a motivational system that informs us of what is important and relevant in our surroundings, and help us navigate and adjust to change (Tomkins, 1962, 1963, 1991; Demos and Tomkins, 1995). Previous research on discrete emotions and affect integration has demonstrated that is essential to differentiate between affects both in theory and assessment, as discrete affects carry specific informational value and motivate specific kinds of behavior (see e.g., Izard, 1991, 2007; Solbakken et al., 2011a; Solbakken and Monsen, 2021; Frederiksen et al., 2022). As such, even though the two integration types *driven* and *lack of access* describe prototypical modes of relating to affects in general, we emphasize that the specific affect in question is an essential aspect of the integration type. E.g., being driven by anger and being driven by shame are very different at both the experiential and expressive level (e.g., anger outbursts vs. excessive shameful rumination), even though both processes involve being overwhelmed by an affect. Individuals struggling with different affects will have different types of problems, as their struggles are based on affects with different phenomenology and functions. Research that does not differentiate between affects fails to account for these differences. Unfortunately, research on individual differences in affective processes rarely differentiates between affects. The studies that have been conducted, however, show that differentiating between affects yields more informative findings, and report this approach as an advantageous focus for future research (Rivers et al., 2007; Holmqvist, 2008; Monsen et al., 2008; Augustine and Hemenover, 2009; Webb et al., 2012; Johansen et al., 2013; Solbakken et al., 2017; Vishkin et al., 2019; Fiskum et al., 2021; Frederiksen et al., 2021a; Solbakken and Monsen, 2021). As such, the explicit focus on discrete affects in the AC model makes integration types a concept we believe is better suited to access the nuances of affective processes than other related but undifferentiated concepts.

### Integration types and interpersonal problems

Affects convey social information, and social processes are systematically related to affective responses (Smith and Weihs, 2019). The way we experience and express our affects are consequently important factors for efficient communication and social coordination (Van Kleef, 2016). It is thus not surprising that research has shown an association between affective functioning (including affect integration) and interpersonal difficulties, both in clinical and non-clinical populations (Wei et al., 2005; Lech et al., 2008; Adrian et al., 2011; Solbakken et al., 2011a,^2012^, ^2017^; Graling, 2013; Herr et al., 2013; Normann-Eide et al., 2013, 2015; Besharat et al., 2014; Keating et al., 2018; Poole et al., 2018; Akyunus et al., 2020; Frederiksen et al., 2021a; Solbakken and Monsen, 2021).

According to the AC model, when an affect is inadequately integrated it can lead to disruption in the function of the affect, and this disruption inform us of what kinds of problems an individual is likely to encounter (Monsen and Solbakken, 2013; Solbakken et al., 2017). As the functions of affects often are related to interpersonal functioning, we can form hypotheses about the associations between low affect integration for specific affects and various forms of interpersonal difficulties, based on the function of the affect in question. E.g., anger is important for self-assertion, and helps us avoid being exploited (Izard, 1991; Ekman, 2004; Solbakken, 2013). Problems in the integration of anger is thus expected to interfere with healthy self-assertion. What exact difficulties an individual experiences in turn depends on their integration type: a person driven by anger will struggle with being overly self-assertive, leading to interpersonal difficulties such as domineering or controlling behavior. A person lacking access to anger, on the other hand, will struggle with self-assertion, leading to interpersonal difficulties such as submissive behavior.

### Aims of the study

In summary, the two integration types *driven* and *lack of access* denote prototypical ways of relating to and dealing with affects. We believe that adding integration types to the AC model will increase the model’s ability to describe individual differences in affective processes. In this study we will (1) examine the internal structure of integration types by performing CFAs that test and compare different models of affect integration, and (2) investigate the external validity of integration types by testing hypotheses about associations between integration types and specific interpersonal problems.

## Materials and methods

### Participants and procedures

In accordance with Norwegian law, studies based on anonymous questionnaire data can be conducted without further ethical approval. A total of 157 participants from a community sample anonymously completed a questionnaire comprising several psychological measures, either at lectures at university or in their own home. All participants gave written and informed consent. The sample consisted of 71.2% females, the majority of which were students. Mean age was 27.4 years (range = 16–90; SD = 15). The participants had completed an average of 14.4 years of education.

### Instruments

#### The Affect Integration Inventory

The Affect Integration Inventory (AII 2.0; Solbakken and Monsen, 2013) is a self-report instrument designed to assess affect integration. It is based on the ACI (Monsen et al., 1996, 2008) and has been validated in numerous previous studies (Solbakken et al., 2017; Solbakken and Monsen, 2021; Frederiksen et al., 2022). It consists of 112 items and measures the integration of nine discrete affects: 1. Interest/Excitement; 2. Enjoyment/Joy; 3. Fear/Panic; 4. Anger/Rage; 5. Shame/Humiliation; 6. Sadness/Despair; 7. Jealousy/Possessiveness; 8. Guilt/Remorse; and 9. Tenderness/Care. Each item is rated on a 10-point Likert scale, ranging from *does not fit at all* (0) to *fits perfectly* (9).

The AII 2.0 is usually analyzed in terms of scores on three separate levels: global affect integration (overall score across all items); affect experience (mean score for capacity for experience across affects) and affect expression (mean score for capacity for expression across affects); and integration of each discrete affect (e.g., mean score for integration of Interest/Excitement, Enjoyment/Joy, etc.).

#### Integration type subscales

The integration type subscales describe the prototypical manners in which individuals relate to various discrete affects, including how they experience and express them. The two integration types operationalized in the AII 2.0 are Driven and Lack of Access, and the scales link each integration type to a discrete affect (e.g., Driven by Anger).

The subscales were not tested in the initial validation of the instrument and are not traditionally available in other procedures for assessing affect integration. They were created by selecting items from the AII 2.0 that theoretically correspond to the relevant integration types. For some affects, there is as little as one item tapping an integration type, whereas for others there are up to five. Additionally, not all the affects have items representing both integration types. Affects with two or more items tapping a given integration type were accepted for inclusion. The scales included in our analyses were the following: Driven by Anger (example item: “I am afraid of losing control over my anger or afraid of what might happen if I get angry”), Lack of Access to Anger (example item: “It is difficult for me to allow myself to feel angry even when I have good reason”), Driven by Guilt (example item: “I feel burdened by too much guilt”), Lack of Access to Guilt (example item: “When I feel guilty about something, I try not to think about it”), Driven by Shame (example item: “Shame and embarrassment cause me to avoid important social contexts”), Lack of Access to Interest (example item: “I feel less interest and excitement than I would like”), and Driven by Jealousy (example item: “When I get jealous, it can grind on and on in my mind without me being able to stop it”).

As the AII 2.0 was developed to measure the broader concept of affect integration, high scores traditionally reflect adaptive functioning and high affect integration. However, the integration types reflect prototypically problematic ways of experiencing affect. Thus, for ease of reading and interpretation, scores have been organized so that high scores on these scales are reflective of increased problem load.

#### Inventory of Interpersonal Problems (IIP-64)

**FIGURE 1:**
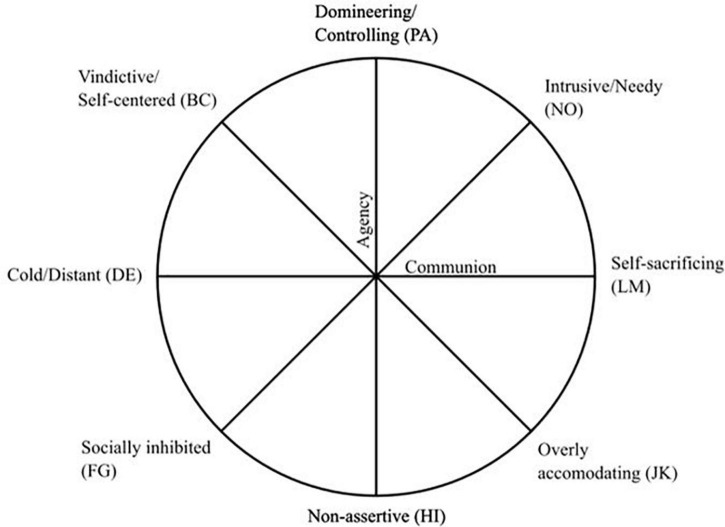
The IIP-64 circumplex, based on Alden et al. (1990) and Horowitz et al. (2000). The eight interpersonal octants denoted counterclockwise starting from the top of the agency axis with the abbreviations PA, BC, DE, FG, HI, JK, LM, and NO.

The IIP-64 measures typical problems arising in interpersonal interactions (Horowitz et al., 1988). In this study the 64-item IIP-circumplex version was used (Alden et al., 1990; Horowitz et al., 2000). Items are phrased either “It is hard for me to.” (39 items) or “These are things I do too much…” (25 items) and are rated on a five-point Likert scale, ranging from *not at all* (0) to *very much* (4). The circumplex structure of the IIP-64 is illustrated in Figure 1. The structure is a result of interpersonal problems being organized along two orthogonal dimensions: agency (non-assertive vs. domineering/controlling) and communion (over-nurturing vs. cold/distant). Taken together, scores on items associated with these two dimensions produce eight subscores (octants in circular space) consisting of eight items each indicating specific problems with being: domineering/controlling (PA),^1^ vindictive/self-centered (BC), cold/distant (DE), socially inhibited (FG), non-assertive (HI), overly accommodating (JK), self-sacrificing (LM), and intrusive/needy (NO). The IIP-64 yields a score for each octant. A score for the overall level of interpersonal problems (IIP-global) is also produced by computing the mean across all 64 items. The present study used a Norwegian version of the IIP-64 translated in 1994 by Stiles and Høglend. This version has been reported to have excellent psychometric properties, comparable to those of the original English version (Monsen et al., 2006). In the present study sample, both the global score and the respective interpersonal subtypes have satisfactory reliability (α = 0.74 or higher).

### Statistical analyses

#### Descriptive statistics and internal consistency

Means, standard deviations, ranges, and estimates of reliability of the different scores are computed. Cronbach’s alpha is used to assess internal consistency reliability. Based on DeVellis (2012), we consider the alpha values either unacceptable (<0.6), acceptable (0.6–0.7), satisfying (>0.7), or excellent (>0.8).

#### Structural validity: Confirmatory factor analysis

In the first part of the study, we employ Structural Equation Modeling (SEM) to conduct CFAs using IBM SPSS Amos to examine whether it is empirically justifiable to distinguish between integration types. We do this by creating and reviewing models for the integration types for each affect individually (e.g., a separate model for Lack of Access to Anger, a separate model for Driven by Jealousy, etc.), as well as four different competing models of affect integration (including various factors loading on all items in the integration type scales).

#### Model specification

In the models of the individual integration type scale**s** an integration type is defined as the latent factor (e.g., Driven by Anger and Lack of Access to Guilt). The indicators are the items in the corresponding AII 2.0 integration type subscale. Some of the integration type subscales consist of only two items, and CFA is not conducted due to inherent model under-identification (i.e., fewer known parameters than freely estimated parameters; Brown, 2015). These include the models for the integration types Driven by Anger, Driven by Guilt, and Driven by Shame.

When investigating the overall internal structure of integration type scales, we compare four competing models of affect integration (all comprising a total of 20 items from the AII) that differ in the proposed structure of affect integration:

Model A: A general affect integration model with “affect integration” as a factor loading on all items.
  Model B: An affect independent integration type model with the integration types “Driven” and “Lack of Access” as factors loading on items in corresponding integration type subscales (indicating integration types that generalize across affects).
  Model C: An affect dependent model with factors for each affect loading on the items for that affect (indicating that affect integration is specific to discrete affects; no integration types).
  Model D: An affect dependent integration type model with affect specific integration types (e.g., Driven by Anger) as factors loading on the items in the corresponding integration type subscale (indicating integration types that are specific for discrete affects, i.e., integration types as outlined in the introduction of this article).

We use the items included in the integration type subscales, but not the remaining items in the AII 2.0, as this allows for optimally realistic comparison of the models.

#### Model selection

The models of the individual integration type scales are evaluated based on the “absolute fit” approach: consulting goodness of fit indices produced by Amos to determine how well the model fits the data. Following Brown’s (2015, pp. 70–75) recommendation, different goodness of fit indices are used, as they provide different information about model fit (absolute fit, parsimony correction, and comparative fit): a standardized root mean square (SRMR) close to or below 0.08 (absolute fit); a root mean square error of approximation (RMSEA) close to or below 0.08 (parsimony correction); a comparative fit index (CFI) and a Tucker–Lewis index (TLI) close to or greater than 0.90 (comparative fit). As indicators of relative fit, we also report Akaike’s information criterion (AIC) and Bayesian information criterion (BIC). Thus, when evaluating the four general models, the absolute fit approach is used, as well as a comparative approach where the models are compared to see which of the models has the best fit. Model fit is also evaluated in all models by examining factor loadings and inspecting the standardized residual covariances to check for localized areas of strain. Standardized residual covariances larger than 2 (or smaller than −2) is considered indicative of localized areas of strain.

#### Research hypotheses regarding CFAs

We hypothesize that the models of individual integration type scales will have adequate model fit, and we expect the scales to have adequate internal consistency. Regarding the competing models of affect integration, we predict that the model with affect specific integration types (Model D) will outperform the other three models and have adequate model fit and internal consistency.

#### Management of missing data and model revision

Missing data are handled by using direct ML in Amos. This allows Amos to present modification indices with suggestions to improve the models. The suggestions are inspected to see if there are theoretical grounds for adding them to the model. Model revisions are done in cases where the modifications suggested are theoretically sound and considerably improves the model. The general models are compared without model revisions, but model revisions are done in the general model that has the best comparative fit.

#### Analyses of nomological criterion related validity

As described in the introduction, we can predict what interpersonal problems are likely to be associated with specific integration types based on the function of the affect in question, as the adaptive signal values of affects often are related to interpersonal functioning. Due to the circular and circumplex composition of the IIP-64, theorems from the mathematics of circle geometry apply. Consequently, distinct sinusoidal patterns of correlations can be predicted with peaks and low points in different and theoretically specified and expected octants of the IIP-64. I.e., we expect an integration type to have a peak association with one particular interpersonal problem [e.g., vindictive/self-centered (BC)], and then gradually lower associations with other interpersonal problems, following the structure of the IIP-64 circumplex [in this example cold/distanced (DE), socially inhibited (FG), etc.], until reaching the interpersonal problem placed directly opposite from the peak correlation in the circumplex [here exploitable/overly accommodating (JK)]. Correlations are expected to increase again from the low point back to the peak correlation, creating a sinusoidal pattern of correlations.

Substantial and significant associations with predicted and separate octant scores for the respective integration type of an affect will serve as support for convergent validity. Small and non-significant associations with opposing octants, together with the theoretically expected rank order of correlations, will serve as support for the discriminant validity of the construct. Systematic variation between the patterns of associations for the two integration types will serve as further support for discriminant validity.

Overall convergent validity of AII integration type scales is tested through investigating associations between scores on integration types and overall level of interpersonal problems (IIP-64 global score). Convergent and discriminant validity are examined through testing correspondences between expected and obtained patterns of associations between specific integration types and scores on the IIP-64. Patterns of Pearson’s correlation coefficients (Pearson’s *r*) are computed for this purpose. Applying Gurtman and Balakrishnan”s (1998) structural summary method^2^ and related goodness of fit-index (GoF), sinusoidal structure and fit of the different patterns of correlations between integration types and IIP-subscales are tested. GoF >0.80 is considered good fit (Gurtman and Pincus, 2003). In line with Zimmermann and Wright (2017), an amplitude of 0.15 or greater is used as an indicator of markedly differentiated correlation profiles, while an amplitude of 0.10 or greater is used to indicate a moderately differentiated profile. Similarly, a mean correlation value of 0.15 or greater is used as an indicator of marked elevation. Additionally, *Z*-tests are conducted as a means of assessing the statistical significance of differences in correlation magnitude between the peaks and low points in the respective correlation patterns. The comparisons are estimated by using an Excel Spreadsheet calculator created by DeCoster and Iselin (2009), based on Steiger (1980).

#### Research hypotheses regarding nomological criterion related validity

First, we predict positive associations between scores on different integration types and overall level of interpersonal problems. Second, we hypothesize distinct sinusoidal patterns of associations between the integration types and different types of interpersonal problems, based on the function of the particular affect examined. A large body of research supports he argument for studying the relationship between affect integration and interpersonal problems with reference to distinct affects, and specific theoretically consistent sinusoidal patterns of associations have been demonstrated for various discrete affects in both clinical and non-clinical samples (see e.g., Solbakken et al., 2011a,^2012^, ^2017^; Normann-Eide et al., 2013; Frederiksen et al., 2021a). A thorough discussion of the adaptive functions of different affects is beyond the scope of this paper, but can be found richly described elsewhere (e.g., Izard, 1991; Darwin and Prodger, 1998; Normann-Eide, 2020). Below we briefly present the relational functions of the affects investigated in the present study, along with what interpersonal problems are posited to arise in relation to the integration types.^3^

#### Anger

Anger is important for protecting the self and others, it helps us avoid being exploited and is important for self-assertion (Izard, 1991; Ekman, 2004; Solbakken, 2013). *Lacking access* to anger would leave the individual out of touch with their need to protect, stand up for, and/or fight for themselves, resulting in a tendency of submissive interpersonal behavior. Lack of Access to Anger thus is expected to have a correlation pattern peaking in the non-assertive (HI) octant and having a low point in the dominant (PA) octant. Being *driven by anger* will generate problems with controlling one’s anger, possibly scaring others, or at least making others anxious or cautious. Additionally, being driven by anger is theoretically parallel with hyper-assertiveness, resulting in difficulties accepting normal social and relational restrictions. Driven by Anger is consequently hypothesized to have a correlation pattern peaking in the dominant (PA) octant and having a low point in the non-assertive (HI) octant.

#### Guilt

Izard (1991) consider guilt as the key affect in terms of development of personal and social responsibility, and the phenomenon of conscience. Guilt motivates us to reduce emotional distress within social relationships by eliciting signs of commitment and caring and rectify wrongdoings (Baumeister et al., 1994). *Lack of access* to guilt is likely to result in a lack of consideration for others and a tendency to not take responsibility for one’s actions (Solbakken, 2013). We thus expect it to have a correlation pattern peaking in the vindictive (BC) octant and having a low point in the exploitable (JK) octant. Being *driven by guilt* would give an internal signal to the individual that they are responsible for the feelings and reactions of others and that they should feel bad for even minor transgressions. This would theoretically contribute to difficulties both with self-assertion, speaking up, or confronting others when necessary and also to being overly responsible for others. In turn, a pattern of submissiveness, passivity and social exploitability is likely to emerge. Being Driven by Guilt is expected to have a correlation pattern peaking in the overly accommodating (JK) octant, with a low point in the vindictive (BC) octant.

#### Shame

Shame is important for social conformity, as being sensitive to others’ opinions is a protection against exclusion from the group (Izard, 1991). It can inform us of our social position by increasing our self-awareness, and guide future behavior to maintain social standing, sending outward signals communicating that we do not believe we are better than others (Normann-Eide, 2020). Being *driven by shame* would make the individual vulnerable to feelings of worthlessness and excessive concern about social evaluation inhibiting both the individual’s agency and communal behavior. Interpersonal problems of withdrawal from, inhibition in, and avoidance of social encounters are thus its theoretically expected correlates. Driven by Shame is thus hypothesized to yield a correlation pattern peaking in the socially avoidant (FG) octant and having a low point in the opposing intrusive (NO) octant.

#### Jealousy

Jealousy communicates that the other is of significant importance to us and serves as a motivation to fight or work for our relationships and prevents a threatening liaison between a rival and a loved one (Solbakken, 2013; Chung and Harris, 2018; Leahy, 2018). Being *driven by jealousy* can be expected to involve an increased need for control, possibly by threatening the significant other (verbally or physically) to remain in the relationship or acting hostile toward those considered rivals. Impulsivity and diminished ability to mentalize might also be plausible. Driven by Jealousy is thus expected to have a correlation pattern peaking in the vindictive (BC) octant, with a low point in the exploitable octant (JK).

#### Interest

Interest guides our attention by helping us prioritize which elements to focus on (Izard, 1991). It spurs observation and interaction with the world and motivates both being active/agentic and social/communal. *Lacking access to interest* involve passivity, a lack of both personal agency and communal drive, impaired ability to share one’s own interest and excitement with others and possibly impaired sensitivity to other people’s excitement and interest. Lack of Access to Interest is consequently hypothesized to have a correlation pattern peaking in the socially avoidant (FG) octant, with a low point in the opposing intrusive (NO) octant.

## Results

### Descriptive statistics

Table 1 shows a summary of means, standard deviations, ranges and estimates of reliability (Cronbach’s alpha) of the different scores derived from the AII and used in the present study. All Cronbach’s alpha estimates for integration type scales indicated satisfactory internal consistency (range: α = 0.67–0.89, median: α = 0.78). Descriptive statistics for IIP-64 can be found in Supplementary material.

**TABLE 1:** Descriptive data and estimates of reliability for the AII.

AII scale	*M*	SD	Range^a^	α
Global AI score^b^	5.72	0.97	3.14–7.85	0.96
**Integration aspects^c^**				
Experience	5.73	0.97	3.06–8.16	0.94
Expression	5.67	1.22	2.30–8.17	0.91
**Integration types^d^**				
**Driven**				
Anger	3.03	2.25	0.0–8.50	0.73
Guilt	4.35	2.80	0.0–9.00	0.89
Shame	3.02	2.34	0.0–9.00	0.67
Jealousy	2.46	2.05	0.0–8.20	0.86
**Lack of access**				
Anger	5.43	1.63	0.75–9.00	0.75
Guilt	6.34	1.46	2.20–9.00	0.78
Interest	5.86	1.70	1.00–9.00	0.80

α, Cronbach’s alpha.

^a^Maximum range: 0–9.

^b^Mean score across integration aspects and affects.

^c^Mean scores for each integration aspects across all affects.

^d^Mean scores for each integration type across integration aspects.

### Structural validity: Confirmatory factor analysis

#### Models of the specific integration type scales

The separately conducted CFAs of the models of the integration type scales revealed that the Lack of Access to Anger and Driven by Jealousy models had satisfactory model fit according to all goodness of fit indices. The models of Lack of Access to Guilt and Lack of Access to Interest had satisfactory model fit according to some, but not all, goodness of fit indices. The modification indices presented by Amos suggested adding correlations between error terms in both models, indicating that the items in question had shared variance not explained by the factor. These modifications did not meaningfully change the theory the models were based on and were judged to be theoretically sound. The resulting correlations between error terms were of varying sizes (0.20, 0.38, and 0.53). After modification, all the models of the integration type scales^4^ had satisfactory goodness of fit according to the goodness of fit indices, as shown in Table 2. Factor loadings were generally high, though with some variation, and are presented in Table 3. All factor loadings were above 0.30. Inspection of the standardized residual covariances revealed no localized areas of strain, indicating good model fit.

**TABLE 2:** Goodness of fit indices for the models of integration type scales.

Model	SRMR	RMSEA (90% confidence intervals)	CFI	TLI
Lack of Access to Anger	0.0248	0.049 (0.000–0.174)	0.995	0.986
Lack of Access to Guilt	0.0082	0.000 (0.000–0.069)	1.000	1.026
Lack of Access to Interest	0.0279	0.058 (0.000–0.145)	0.992	0.981
Driven by Jealousy	0.0398	0.084 (0.028–0.137)	0.973	0.955

SRMR, standardized root mean square residual; RMSEA, root mean square error of approximation; CFI, comparative fit index; TLI, Tucker–Lewis index (aka. non-normed fit index).

**TABLE 3:** Factor loadings for the models of integration type scales.

Model	Indicator	Factor loading
Lack of Access to Anger	Ang3	0.656
Lack of Access to Anger	Ang4	0.834
Lack of Access to Anger	Ang5	0.737
Lack of Access to Anger	Ang6	0.462
Driven by Jealousy	Jeal1	0.750
Driven by Jealousy	Jeal2	0.710
Driven by Jealousy	Jeal3	0.812
Driven by Jealousy	Jeal4	0.661
Driven by Jealousy	Jeal5	0.495
Driven by Jealousy	Jeal6	0.777
Lack of Access to Interest	Int1	0.820
Lack of Access to Interest	Int2	0.744
Lack of Access to Interest	Int3	0.799
Lack of Access to Interest	Int4	0.322
Lack of Access to Interest	Int5	0.648
Lack of Access to Guilt	Guil3	0.828
Lack of Access to Guilt	Guil4	0.325
Lack of Access to Guilt	Guil5	0.916
Lack of Access to Guilt	Guil6	0.594
Lack of Access to Guilt	Guil7	0.475

Indicator names signify the affect targeted in the item, e.g., “Ang1” is an item from the AII 2.0 targeting integration of anger.

#### Competing overall models of affect integration

**FIGURE 2:**
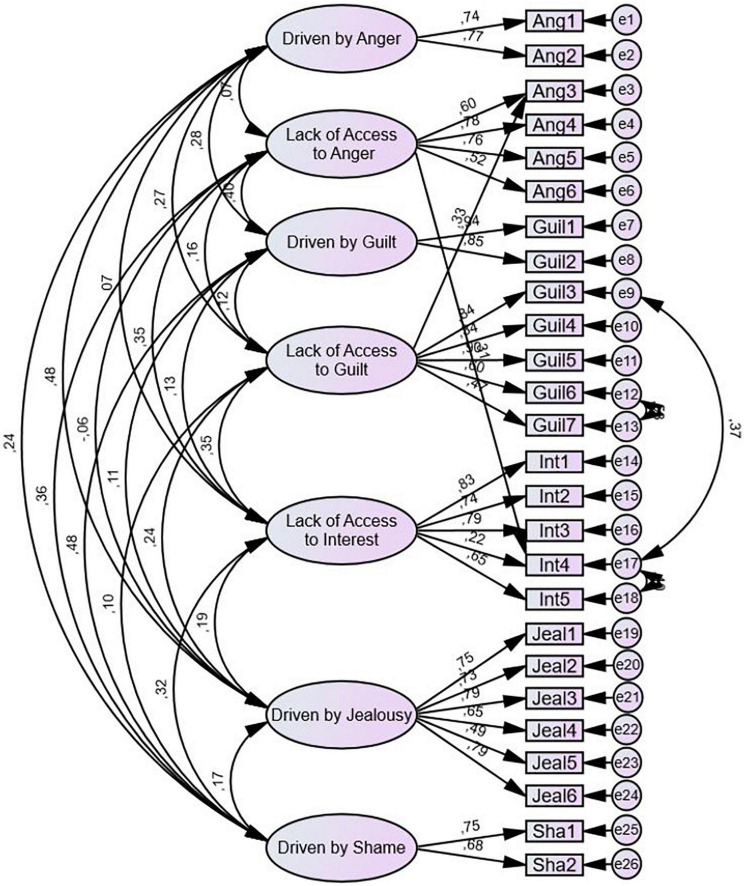
Factor structure of Model D, an affect dependent integration type model with affect specific integration types as factors loading on the items in the corresponding integration type subscale (indicating integration types that are specific for discrete affects). Indicator names signify the affect targeted in the item, e.g. “Ang1” is an item from the AII 2.0 targeting integration of anger. Error terms are labelled “e1”, “e2”, etc.

In line with our predictions, CFAs of the overall models revealed that Model D had the best model fit compared to the other models both in terms of higher factor loadings and the goodness of fit indices.^5^ The latent factor structure and factor loadings in the different models can be found in Supplementary material. Table 4 shows the goodness of fit indices for each model. The model with best fit, Model D, met the cut-off criteria for the SRMR and the RMSEA, but not the CFI or the TLI. After consulting the modification indices generated by Amos, four model revisions were made. These included three added correlations between error terms and two cross loadings: from “Lack of Access to Anger” and from “Lack of Access to Guilt.” The revised model is presented in Figure 2. After revision, the model met the cut-off-criteria for all the goodness of fit indices. Goodness of fit indices are presented in Table 4 along with the indices for the other models. Factor loadings were generally high, varying between 0.22 and 0.94 (of the 28 factor loadings, only 6 were below 0.50; see Supplementary Table 2). Correlations between factors were generally small, supporting the notion that the integration types within and between affects are separate constructs. Despite overall good fit, the standardized residual covariances indicated some localized areas of strain, meaning that there are some relationships between specific variables the model failed to reproduce adequately (see Supplementary material).

**TABLE 4:** Goodness of fit indices for the different models.

Model	SRMR	RMSEA (90% confidence intervals)	CFI	TLI	AIC	BIC
Model A – general affect integration	0.151	0.156 (0.148–0.164)	0.305	0.244	1,592.134	1,624.785
Model B – general integration type	0.139	0.135 (0.127–0.143)	0.482	0.435	1,302.944	1,336.013
Model C – affect dependent integration	0.121	0.105 (0.097–0.114)	0.694	0.656	965.360	1,002.198
Model D – affect specific integration type	0.082	0.074 (0.064–0.084)	0.855	0.830	713.704	713.704
Model D revised	0.072	0.054 (0.042–0.066)	0.923	0.909	606.658	606.658

SRMR, standardized root mean square residual; RMSEA, root mean square error of approximation; CFI, comparative fit index; TLI, Tucker–Lewis index (aka. non-normed fit index); AIC, Akaike’s information criterion; BIC, Bayesian information criterion.

### External criterion validity: Patterns of association between integration type and interpersonal problems

#### Associations with the overall level of interpersonal problems

Table 5 shows the obtained correlations between integration type scales and the overall level of interpersonal problems. The results were in line with our expectations, as all correlations were significant at a 0.01-level and of moderate magnitude (except Driven by Anger). Lack of Access to Anger had a somewhat higher association with the overall level of interpersonal problems than Driven by Anger. For guilt, on the other hand, the association with interpersonal problems was higher for being driven by the affect than lacking access to it. Of all the integration types investigated, being Driven by Shame had the highest correlation with overall level of interpersonal problems, closely followed by Lack of Access to Interest. Both approached large magnitudes according to Cohen’s classification. Being Driven by Anger had the lowest association with overall interpersonal problems.

**TABLE 5:** Correlations between overall interpersonal problems and integration type of different affects.

Integration type	IIP global^a^
**Driven by**	
Anger	0.252**
Guilt	0.408**
Shame	0.464**
Jealousy	0.334**
**Lack of access to**	
Anger	0.337**
Guilt	0.312**
Interest	0.422**

^a^Score of overall interpersonal problems.

**p < 0.01 (two-tailed).

#### Convergent and discriminant associations with specific types of interpersonal problems

**FIGURE 3:**
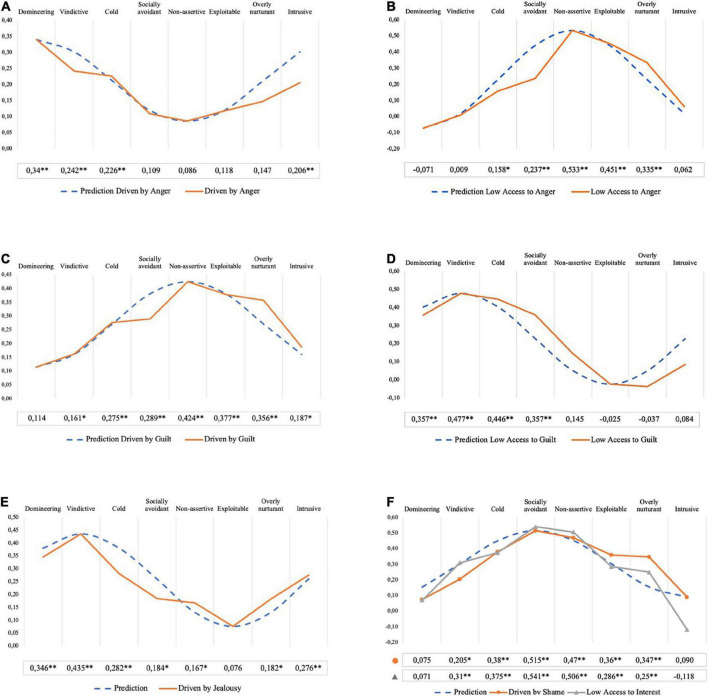
Predicted and obtained correlation patterns for integration types and interpersonal problems. **(A)** Driven by Anger. **(B)** Low Access to Anger. **(C)** Driven by Guilt. **(D)** Low Access to Guilt. **(E)** Driven by Jealousy. **(F)** Driven by Shame and Low Access to Interest.

On the level of specific interpersonal problems and integration types across affects, distinct correlation patterns both *within* and *between* different affects were expected, depending on the integration type in question. Figures 3A–F shows the predicted and obtained correlation patterns for the respective integration types across affects. Table 6 shows results of structural summary analyses of the correlation profiles along with observed peak and low points across all correlation patterns. Sinusoidal patterns of correlations peaked in expected octants of the IIP-64 for all the integration type scales across affects. The peak correlation was significant at 0.01-level in all cases, while all low points were non-significant, as is expected for associations within a sinusoidal pattern. The structural summary analyses provided further support for our hypotheses, with all patterns having elevation above 0.15, and all amplitudes exceeding the threshold value for marked differentiation (0.15) except one (Driven by Anger – which had an amplitude of 0.11, i.e., moderately differentiated). Structural summary-derived angular peak displacements were in the expected octants or slightly rotated clockwise. Thus, as expected, Driven by Anger was placed in PA, Lack of Access to Anger in HI, Driven by Guilt in JK, Driven by Jealousy in BC, Lack of Access to Interest in FG. The remaining scales were slightly rotated counterclockwise in terms of angular displacement; thus, Driven by Shame was placed in HI, rather than FG and Lack of Access to Guilt in DE, rather than BC. As can be readily seen, all patterns had GoF-statistics that exceeded the threshold of good fit (0.80) with the hypothesized pattern, demonstrating a high level of profile prototypicality in line with our expectations. All comparisons of peaks and low points within each correlational pattern were statistically significant at the *p* < 0.01 level, except for “Driven by Anger” which was statistically significant at the *p* < 0.05 level. Supplementary Table 4 details the *Z* scores and *p*-values.

**TABLE 6:** Structural summary statistics and peak and low point correlations across correlation patterns.

Integration type	*e*	*a*	θ	GoF	Peak	Through
D_Anger	0.18	0.11	103°	0.95	PA (*r* = 0.34)	HI (*r* = 0.09)
L_Anger	0.21	0.28	289°	0.95	HI (*r* = 0.53)	PA (*r* = −0.07)
D_Guilt	0.27	0.15	297°	0.91	HI (*r* = 0.42)	PA (*r* = 0.12)
L_Guilt	0.23	0.27	160°	0.96	BC (*r* = 0.48)	LM (*r* = −0.04)
D_Jealousy	0.24	0.15	120°	0.99	BC (*r* = 0.44)	JK (*r* = 0.08)
D_Shame	0.31	0.21	254°	0.92	FG (*r* = 0.52)	PA (*r* = 0.08)
L_Interest	0.31	0.27	235°	0.87	FG (*r* = 0.54)	NO (*r* = −0.12)

*e*, elevation; *a*, amplitude; θ, displacement in degrees from zero (LM); GoF, goodness of fit (*R*^2^) with a cosine curve peaking in the hypothesized octant; peak, highest observed correlation; through, lowest observed correlation; D_Anger, driven by anger; L-Anger, lack of access to anger; D_Guilt, driven by guilt; L_Guilt, lack of access to guilt; D_Jealousy, driven by jealousy; D_Shame, driven by shame; L_Interest, lack of access to interest; PA, domineering; HI, non-assertive; BC, vindictive; LM, overly nurturant; JK, exploitable; FG, socially inhibited; NO, intrusive.

## Discussion

The present study introduced and examined the construct validity of a subdomain of affect integration – integration types. Analyses consisted of CFAs with structural equation modeling, analyses of associations between integration types and overall level of interpersonal difficulties, and tests of hypothesized patterns of associations between integration types and different interpersonal problems. Results demonstrated satisfactory inter-item reliability for all integration type subscales, indicating high internal consistency of scale scores. Confirmatory factor analyses (CFA) supported the internal structure of affect specific integration types. The CFA models for each integration type scale had satisfactory fit, and the overall model specifying integration types across discrete affects (Model D) outperformed all competing models of affect integration. Although some localized areas of strain indicated that there were relationships between specific variables the model failed to reproduce adequately, overall fit of the model with integration types (Model D) was satisfactory based on both GoF-indicators and factor loadings.

Analyses of nomological criterion validity demonstrated moderately strong correlations between integration types and overall level of interpersonal problems, in line with our predictions. All the examined integration types had correlations in line with the characteristic rank order of a sinusoidal pattern (i.e., a peak in the expected octant, with gradually lower correlations toward the low point) and structural summary parameters supported this fact. Convergent validity for differentiating between integration types across affects was supported by finding correlation patterns for the integration type scores having substantial peak-correlations in theoretically expected and separate octants of the IIP-64. Discriminant validity was demonstrated by low and non-significant correlations with octants placed opposite to the expected peak correlation. For the affects where both integration types were examined, peak-correlations were found in diametrically opposite octants, supporting the theoretical notions of the motivational and signal properties of different affects. Mathematically stringent and combined tests of convergent and discriminant validity were provided by obtaining high goodness of fit scores (GoF ≥0.85) with the hypothesized sinusoidal patterns of associations (Gurtman and Balakrishnan, 1998; Gurtman and Pincus, 2003). Testing patterns of associations in this way and obtaining a measure of convergent and discriminant validity simultaneously is commonly considered the optimal method for demonstrating criterion related validity (see e.g., Campbell and Fiske, 1959).

Taken together, these findings appear to highlight that differences in prototypical ways of experiencing and expressing affects:

- Can be assessed easily, quickly, and reliably.
- Have theoretically consistent intra-domain relationships and valid structural psychometric properties.
- Are robustly related to interpersonal functioning in general.
- Are systematically and differentially related to specific and theoretically predictable interpersonal problem types.

Our results give proof of concept that adding integration types as a construct contributes to elaborating the AC framework and can delineate an important aspect of how affect integration varies between individuals. It provides a description of how difficulties in affect integration can manifest differently based on how the individual relates to their affects, i.e., whether they are driven by or lack access to them. Affect integration theory emphasizes that we use the information from our affects to evaluate the situation and guide our behavior. Having adequate levels of affect integration for different affects involves being able to use these signals in a flexible way, enabling the individual to adaptively interact with their social environment. When an individual has low levels of AC, however, this ability is impaired and the manner in which the individual relates to their affects (i.e., integration type) shapes their interaction with the social environment in theoretically predictable ways based on the function of the affect.

This theoretical conjecture is supported empirically in this study, as the associations between integration types and interpersonal problems were in line with our predictions. Additionally, CFAs show that incorporating integration types into the AC framework accounts for our empirical data in a more informative way than the more general models, thereby capturing nuances of affective processes that the present AC model does not incorporate. The results are in line with and add further nuance to previous findings of associations between affect integration of specific affects and interpersonal problems (Solbakken et al., 2011a,^2012^, ^2017^; Normann-Eide et al., 2013; Frederiksen et al., 2021a; Solbakken and Monsen, 2021), as well as findings that emotion regulation is associated with and in many cases mediate interpersonal processes (Wei et al., 2005; Adrian et al., 2011; Graling, 2013; Herr et al., 2013; Besharat et al., 2014; Keating et al., 2018; Poole et al., 2018; Akyunus et al., 2020).

Although integration types are specific to affect integration theory, our results suggest that more elaborate models of affective processes have greater explanatory value than general models and allow for better theoretical descriptions of complex processes. Theoretical models of affective processes would benefit from incorporating more nuanced concepts like the integration types proposed here. This includes both an explicit focus on the importance of discrete affects and using concepts that describe how affective processes can unfold in different manners (rather than a singular focus on whether individuals relate to their affects in an adaptive or maladaptive way). Methodologically, operating with more specific concepts allows researchers to access variation that might be masked when studying general concepts. This enables us to access processes that have so far remained uncovered or untested, thereby furthering our understanding of psychological phenomena and how they are related to each other.

A better understanding of how psychological phenomena unfold can have important practical consequences as it informs theory development, clinical practice and policy making. For instance, although the community sample in this study is non-clinical, we believe that incorporating integration types in the AC model will have important clinical implications as well. The AC framework emphasizes that low affect integration is at the core of most psychiatric disorders (Monsen et al., 1996). This stance is supported through studies demonstrating that therapy targeting affect integration in patients with various psychological problems including the presence of personality disorders is effective (Monsen et al., 1995; Solbakken et al., 2012). As targeting affect integration in general has yielded highly promising results, it is plausible that we would see the same for therapy specifically targeting integration types. The results in the present study can guide the practitioner with regard to what interpersonal problems to be aware of when a patient describes emotional problems (and vice versa), allowing for even more tailored interventions.

### Limitations

First, due to the correlational and cross-sectional design of the study, no conclusions can be drawn regarding causality. Although the theoretical framework presented generally argues that integration types have a causal effect on social outcomes, it is also a possibility that interpersonal problems have reciprocal effects on, or even precede, affective difficulties (see e.g., Horowitz et al., 2006). Second, the fact that both affect integration and interpersonal problems were self-reported increases the likelihood of inflated associations due to common method variance. Third, the data in this study have been used in previous research investigating the relationship between affect integration and interpersonal problems and somatization (Lødrup and Rauk, 2015; Solbakken et al., 2017; Solbakken and Monsen, 2021). Conducting several analyses using the same dataset increases the probability of making a type-I error, and future research should attempt to replicate the results. Additionally, the analyses of the relationships between integration types and IIP-64 in the present study are secondary (see e.g., Heaton, 2008), as Solbakken et al. (2017) investigated similar associations (though not integration types). The method of secondary analysis allows the investigation of other topics than those of the initial studies, opening the possibility for new insights. However, it demands that the researchers are open and attentive when presenting and interpreting the results, as previous findings are likely to affect how the present results are understood (von der Lippe et al., 2019). The first and second author were not involved in previous research with the same data material, which is an advantage in this circumstance.

Fourth, some characteristics of the sample should be noted when considering the generalizability of the results. As the data were drawn from a community sample it remains unclear whether the findings also apply to clinical settings and specific patient groups. Furthermore, it may be that the sample is not entirely representative, as it consists of mostly students and has a large percentage of females and people with higher education. However, previous studies on the same sample have controlled for both sex and age, without finding any substantial contribution of these factors (Solbakken et al., 2017).

Lastly, we only investigated integration types for five discrete affects. Out of these, only two affects (anger and guilt) were examined in regard to both integration types. Ideally, we would have explored both integration types with regard to all of the affects. Due to the composition of the version of the AII used in this study, this was not possible. Additionally, the integration types examined were primarily coupled with unpleasant affects, except for Lack of Access to interest. Thus, this study does not examine being driven by any pleasant affect. It might, e.g., be that being driven by pleasant affects is not associated with interpersonal problems to the same degree as being driven by negative affects, as experiencing pleasant affects are generally associated with positive outcomes (e.g., see Pressman et al., 2019). Nonetheless, the findings in this study represent a promising start in terms of uncovering the differences between integration types and understanding the nuances in the AC construct.

### Recommendations for future research

To gain a more comprehensive understanding of integration types, more research is needed. It would be beneficial to examine both integration types across all affects with regard to how they relate in the interpersonal space. Recently, a new as of yet untested version of the AII was created with integration type scales for all affects, which can be used for this purpose (the AII 3.2; Solbakken and Monsen, 2020).

Investigating how integration types are associated with various other external criteria would be important for further determining external validity. Examining how integration types relate to measurements of psychological distress and psychiatric symptoms (e.g., OQ-45 and SCL-90) would broaden the theoretical understanding of the integration type construct and possibly assist in determining where to target interventions in clinical settings. Additionally, it would be interesting to investigate how integration types relate to other constructs that measure affective functioning, such as emotion regulation strategies and alexithymia. Previous studies have indicated a close link between global affect integration and these phenomena (Solbakken et al., 2017). Specifically looking at the integration types would aid in discovering more nuanced differences and similarities between the different constructs, as well as between the two integration types.

It would also be beneficial to investigate how integration types are associated with interpersonal problems in diverse clinical samples. Exploring various patient populations may help determine whether different disorders are characterized by one integration type or the other, or if the difficulties are better captured by measuring overall affect integration. This would add to previous studies suggesting that low affect integration for different groups of affects is related to different personality disorders (Johansen et al., 2013; Frederiksen et al., 2021b) and different types of interpersonal problems in patients with personality disorders (Normann-Eide et al., 2013).

Another interesting line of inquiry is further investigating the nature of the integration types. One possible course is to study how patterns of integration types manifest in individuals. Individuals might have a tendency toward being driven by or lacking access to all (or most) affects, or the integration type for each affect might be uncorrelated. Integration types might interact, influencing the manifestation of each other. Other psychological factors might influence what integration type is manifested; further research could, e.g., investigate attachment, personality traits and cognition, as these are associated with affective processes (e.g., Wei et al., 2005; Storbeck and Clore, 2007; Augustine et al., 2013; Joormann, 2019; Segerstrom and Smith, 2019; Hughes et al., 2020).

Lastly, one could examine where in the affect integration process the integration type is determined. For instance, having low access to an affect can occur because a person fails to recognize the affect, they recognize, but do not tolerate the affect, or they do not communicate the affect successfully. A person struggling with recognizing and understanding their affects might have different and possibly more severe problems than a person who understands what they are feeling but struggles with effective communication. It could be interesting to examine whether integration types stemming from different steps in the affect integration process are differentially associated with specific problems or problem severity. This would enhance the theoretical understanding of the integration type construct and affective functioning in general, as well as having clinical utility.

## Data availability statement

The data analyzed in this study is subject to the following licenses/restrictions: The data are not publicly available due to privacy restrictions, as participants did not agree to their data being publicly shared. Requests to access these datasets should be directed to OS, o.a.solbakken@psykologi.uio.no.

## Ethics statement

Ethical review and approval was not required for this study on anonymous questionnaire data provided by human participants in accordance with the local legislation and institutional requirements. The patients/participants provided their written informed consent to participate in this study.

## Author contributions

AV and MS: conceptualization, methodology, validation, formal analysis, writing—original draft, and writing—review and editing. OS: conceptualization, methodology, investigation, resources, data curation, writing—original draft, writing—review and editing, supervision, project administration, and funding acquisition. All authors contributed to the article and approved the submitted version.

## Footnotes

1 The interpersonal circumplex is commonly divided into eight octants and denoted in a counterclockwise fashion starting from the upper part of the agency axis with the abbreviations PA, BC, DE, FG, HI, JK, LM, and NO.

2 As per Zimmermann and Wright (2017), predicted patterns of associations can be formally represented by the following cosine function equation: r*^p^*_*i*_ = e + a (cos(θ_*i*_-δ)) where r*^p^*_*i*_ is a construct’s predicted correlation with octant *i*, *e* is the elevation of the profile (the average correlation across all octants), *a* is the amplitude of the profile (the distance between the average correlation and the peak correlation), θ*_*i*_* is the angle of octant *i*, and δ is the angular displacement of the peak of the profile from 0°. Goodness of fit to the expected pattern, or R^2^, is calculated with the formula: R^2^ = Σ^8^*_*i = 1*_* (*r*^p^*_*i*_*-r*^p^*)^2^/Σ^8^*_*i=1*_* (*r*^o^*_*i*_* – *r^o^*)^2^ = SS^p^/SS^o^ where SS is the sum of squared deviations from the mean of the profile for predicted (^p^) and observed (^o^) scores respectively. *R*^2^ can be interpreted as the level of fit to a cosine curve, often termed prototypicality.

3 We present hypothesized patterns and the theoretical rationale for the integration types that are examined in the study. For anger and guilt, both integration types are investigated. For interest, lack of access will be investigated. For shame and jealousy, being driven will be examined.

4 The scales Driven by Anger, Driven by Guilt, and Driven by Shame are not included, as they had only two items and CFA was not possible to conduct.

5 Including AIC and BIC-values.
