# Supplementary material for: Driven or lacking access: Integration types as a subdimension of the affect consciousness construct
Source: Front Psychol. 2023 Feb 15;14:968737. doi: 10.3389/fpsyg.2023.968737 (PMC9977065; doi:10.3389/fpsyg.2023.968737)
Supplement: Supplementary file 1 [file Data_Sheet_1.docx]

Supplementary Material

##
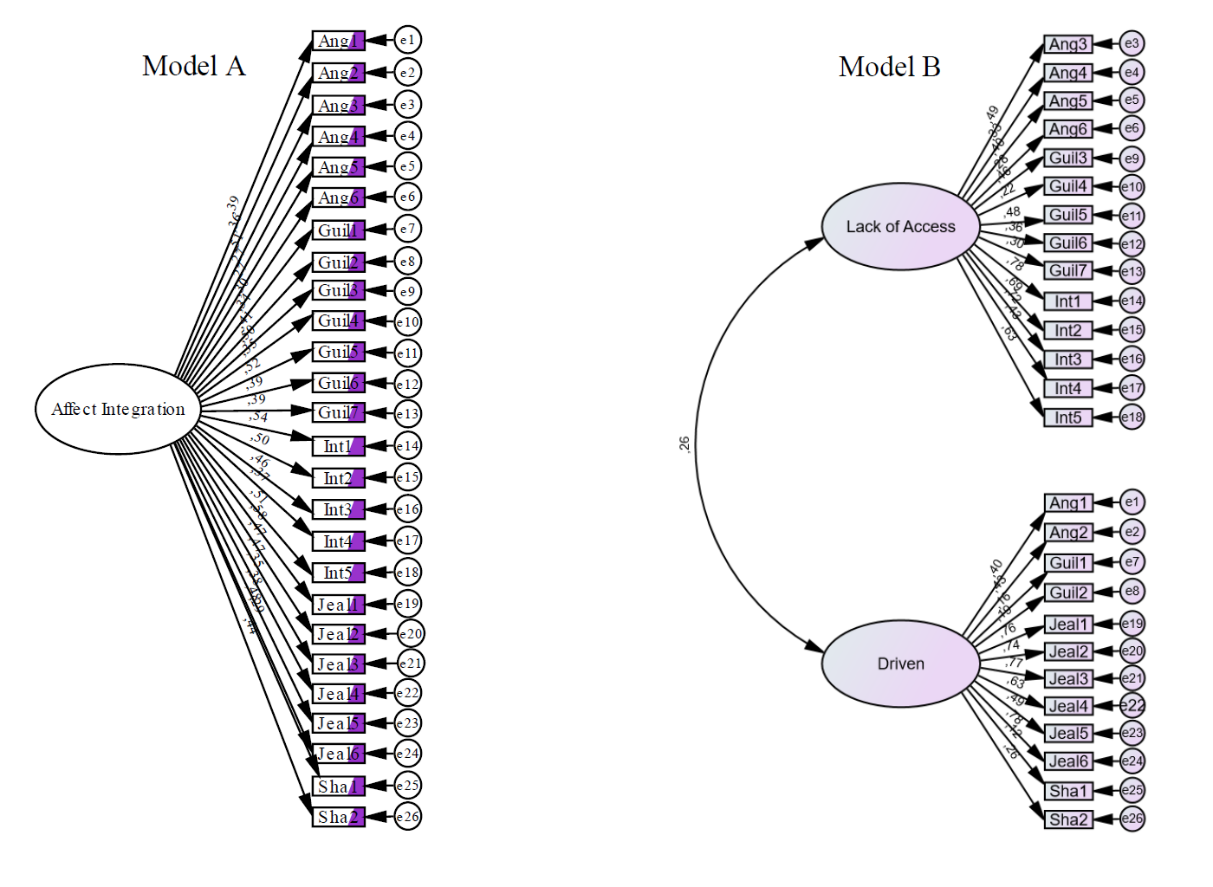
Supplementary Figure


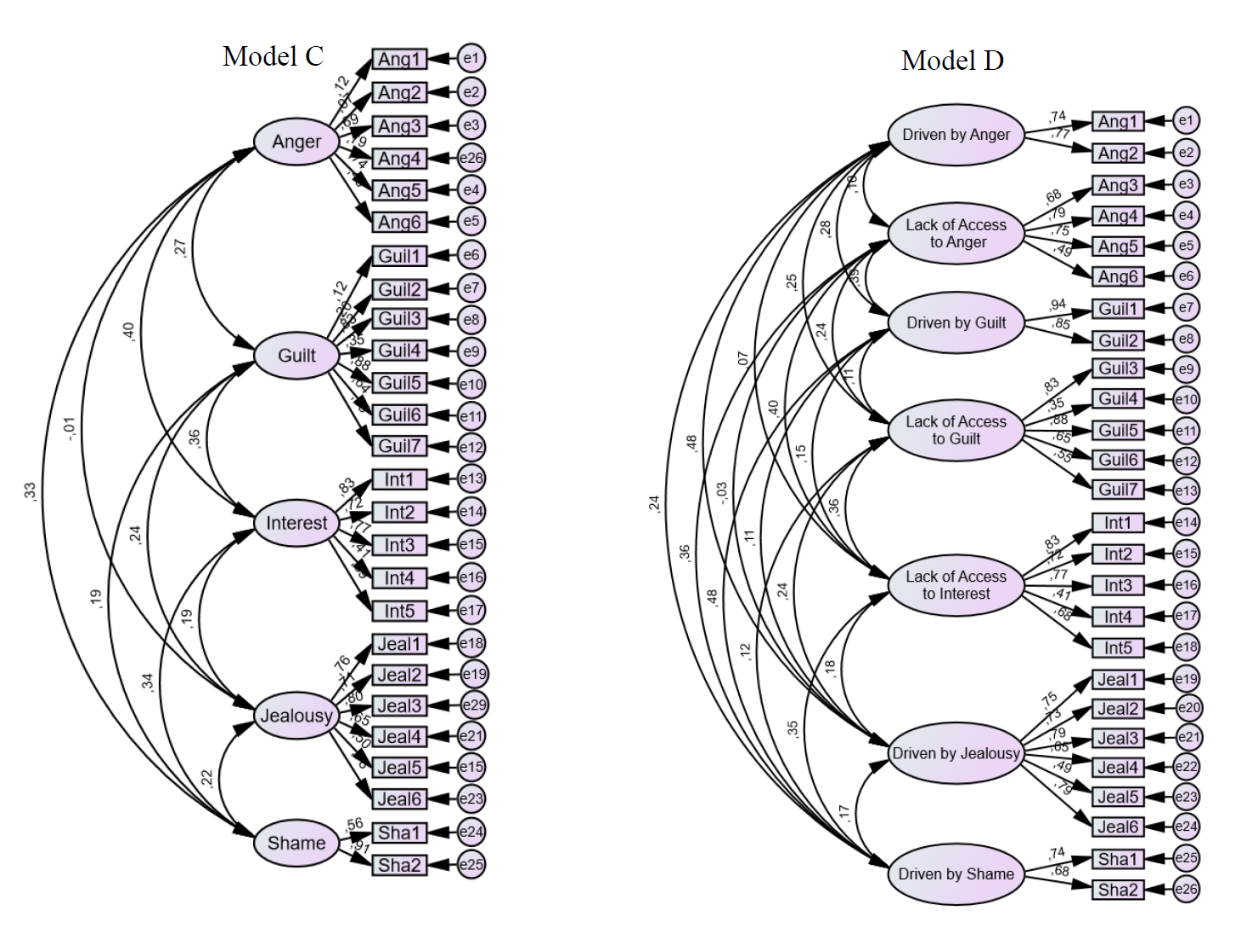


**Supplementary Figure 1.** Factor structure of the four competing models of affect integration.

# Supplementary Tables

**Table 1**

*IIP-64, descriptive data and estimates of reliability*

| IIP-64 | M | SD | Range | α |
| --- | --- | --- | --- | --- |
| Global | 1.00 | .46 | 0.16 – 2.39 | .94 |
| Interpersonal subtypes |  |  |  |  |
| PA - Domineering | 0.68 | .54 | 0.00 – 3.63 | .75 |
| BC - Vindictive | 0.65 | .54 | 0.00 – 2.63 | .75 |
| DE - Cold | 0.75 | .63 | 0.00 – 3.00 | .81 |
| FG - Socially inhibited | 1.06 | .80 | 0.00 – 3.75 | .87 |
| HI – Non-Assertive | 1.26 | .76 | 0.00 – 3.50 | .84 |
| JK – Overly  Accommodating | 1.20 | .63 | 0.00 – 3.50 | .75 |
| LM - Self-sacrificing | 1.44 | .72 | 0.00 – 3.75 | .79 |
| NO - Intrusive | 0.97 | .65 | 0.00 – 2.88 | .74 |

*Note*:M = mean, SD = standard deviation, α = estimate of reliability with Cronbach’s alpha

**Table 2**

*Factor loadings in Model D revised.*

| Factor | Indicator | Factor loading |
| --- | --- | --- |
| Driven by Anger | Ang1 | .743 |
| Driven by Anger | Ang2 | .765 |
| Lack of Access to Anger | Ang3 | .599 |
| Lack of Access to Anger | Ang4 | .783 |
| Lack of Access to Anger | Ang5 | .758 |
| Lack of Access to Anger | Ang6 | .521 |
| Driven by Guilt | Guil1 | .937 |
| Driven by Guilt | Guil2 | .846 |
| Lack of Access to Guilt | Guil3 | .836 |
| Lack of Access to Guilt | Guil4 | .337 |
| Lack of Access to Guilt | Guil5 | .904 |
| Lack of Access to Guilt | Guil6 | .596 |
| Lack of Access to Guilt | Guil7 | .472 |
| Lack of Access to Interest | Int1 | .827 |
| Lack of Access to Interest | Int2 | .74 |
| Lack of Access to Interest | Int3 | .791 |
| Lack of Access to Interest | Int4 | .218 |
| Lack of Access to Interest | Int5 | .655 |
| Driven by Jealousy | Jeal1 | .752 |
| Driven by Jealousy | Jeal2 | .728 |
| Driven by Jealousy | Jeal3 | .793 |
| Driven by Jealousy | Jeal4 | .654 |

| Factor | Indicator | Factor loading |
| --- | --- | --- |
| Driven by Jealousy | Jeal5 | .489 |
| Driven by Jealousy | Jeal6 | .787 |
| Driven by Shame | Sha1 | .749 |
| Driven by Shame | Sha2 | .678 |
| Lack of Access to Guilt | Ang3 | .331 |
| Lack of Access to Anger | Int4 | .31 |

*Note:* Indicator names signify the affect targeted in the item, e.g. “Ang1” is an item from the AII 2.0 targeting integration of anger.

**Table 3**

*Standardized Residual Covariances for model D.*

| Indicator 1 | Indicator 2 | Standardized residual covariance (z-scores) |
| --- | --- | --- |
| Int5 | Sha2 | 2.69 |
| Int5 | Guil2 | 2.32 |
| Sha2 | Int4 | 2.39 |
| Sha1 | Jeal4 | -2.70 |
| Guil2 | Jeal1 | 2.16 |
| Guil2 | Int4 | 2.29 |
| Guil1 | Guil6 | -2.12 |
| Guil1 | Ang6 | 2.21 |
| Ang1 | Ang6 | 2.41 |
| Jeal5 | Guil4 | 2.06 |
| Jeal4 | Guil4 | 2.34 |
| Jeal3 | Guil4 | 2.61 |
| Jeal2 | Guil4 | 3.18 |
| Jeal1 | Ang3 | 2.52 |

*Note*. Only residuals larger than 2.0 is reported. Indicator names signify the affect targeted in the item, e.g. “Ang1” is an item from the AII 2.0 targeting integration of anger.

**Table 4**

*Comparison of correlation coefficients.*

| Integration type | Z | p-value (two-tailed) |
| --- | --- | --- |
| Driven by Anger | 2,500 | 0.012 |
| Lack of Access to Anger | 6,330 | <.000 |
| Driven by Guilt | 3,139 | 0.0017 |
| Lack of Access to Guilt | 5,770 | <.000 |
| Driven by Interest | 5,235 | <.000 |
| Lack of Access to Interest | 6,597 | <.000 |
| Driven by Jealousy | 3,645 | <.001 |
| Driven by Shame | 5,319 | <.000 |
